# Evolution of the assassin’s arms: insights from a phylogeny of combined transcriptomic and ribosomal DNA data (Heteroptera: Reduvioidea)

**DOI:** 10.1038/srep22177

**Published:** 2016-02-26

**Authors:** Junxia Zhang, Eric R. L. Gordon, Michael Forthman, Wei Song Hwang, Kim Walden, Daniel R. Swanson, Kevin P. Johnson, Rudolf Meier, Christiane Weirauch

**Affiliations:** 1 Department of Entomology, University of California, Riverside, Riverside, CA 92521, USA; 2 Lee Kong Chian Natural History Museum, Department of Biological Sciences, National University of Singapore, 117377, Singapore; 3 Illinois Natural History Survey, Prairie Research Institute, University of Illinois, Champaign, IL 61820, USA

## Abstract

Assassin bugs (Reduvioidea) are one of the most diverse (>7,000 spp.) lineages of predatory animals and have evolved an astounding diversity of raptorial leg modifications for handling prey. The evolution of these modifications is not well understood due to the lack of a robust phylogeny, especially at deeper nodes. We here utilize refined data from transcriptomes (370 loci) to stabilize the backbone phylogeny of Reduvioidea, revealing the position of major clades (e.g., the Chagas disease vectors Triatominae). Analyses combining transcriptomic and Sanger-sequencing datasets result in the first well-resolved phylogeny of Reduvioidea. Despite amounts of missing data, the transcriptomic loci resolve deeper nodes while the targeted ribosomal genes anchor taxa at shallower nodes, both with high support. This phylogeny reveals patterns of raptorial leg evolution across major leg types. Hairy attachment structures (fossula spongiosa), present in the ancestor of Reduvioidea, were lost multiple times within the clade. In contrast to prior hypotheses, this loss is not directly correlated with the evolution of alternative raptorial leg types. Our results suggest that prey type, predatory behavior, salivary toxicity, and morphological adaptations pose intricate and interrelated factors influencing the evolution of this diverse group of predators.

Reduvioidea (>7,000 spp.) are the most diverse superfamily of predatory insects and contain a medically important group (~140 spp.) of vertebrate blood feeders^1^,^2^, the Chagas disease transmitting Triatominae, or kissing bugs^3^. Reduvioidea, the assassin bugs, comprise the speciose Reduviidae (~7,000 spp.) and the small and cryptic Pachynomidae (20 spp.)^1^,^2^,^4^,^5^. Prey patterns within Reduvioidea range from euryphagous to stenophagous, with members of some taxa feeding on various arthropods while others are specialized on certain groups, i.e., millipedes (Ectrichodiinae^6^), termites (Salyavatinae and some Harpactorinae^7^,^8^,^9^), ants (Holoptilinae^10^) and spiders (Emesinae^11^). This group has also evolved diverse morphological and behavioral adaptations for prey capture that include various modifications of the forelegs^6^,^11^,^12^,^13^,^14^,^15^,^16^,^17^,^18^, such as hairy attachment structures known as the “fossula spongiosa”, chelate or subchelate forelegs, elaborate armature, and the application of sticky substances (“sticky traps”). These leg modifications are thought to be important for tightly gripping prey before the toxic saliva takes its immobilizing effect^13^,^16^,^18^,^19^,^20^,^21^,^22^,^23^,^24^ (Fig. 1). We here collectively refer to such modified forelegs as raptorial legs.

The fossula spongiosa, a cushion-like expanded area ventrodistally on the tibia composed of thousands of minute tenent hairs covered in an oily secretion, is widespread amongst Reduvioidea and often portrayed as the “typical” assassin bug raptorial leg. This structure is very large in some groups (e.g., Peiratinae; Fig. 1B), but absent or small in others (e.g., Emesinae, Harpactorinae)^18^,^25^,^26^,^27^,^28^. Multiple losses of the fossula spongiosa within the Reduvioidea have been postulated^27^, and it has been suggested that these losses may be correlated with the evolution of alternative raptorial legs^18^,^22^ (also see Fig. S3).

Recent studies on the phylogeny of assassin bugs have made great advances using multilocus or morphological data, and the monophyly of many of the 27 reduvioid subfamilies and certain larger clades are now corroborated^2^,^5^,^20^,^22^,^27^,^29^. However, the backbone of the Higher Reduviidae, a clade comprising most of the assassin bug diversity (~6,500 spp.), is still poorly resolved and weakly supported^2^. This leaves us with limited understanding of the overall phylogenetic relationships of assassin bugs. Interpretations of character evolution, such as those on the evolution of raptorial legs, have therefore remained tentative.

Next-generation sequencing technology has produced a wealth of genomic data for phylogenomic analyses, which now shed light on parts of the Tree of Life that were previously intractable based on morphology or molecular analyses using few loci^30^,^31^,^32^,^33^. Integrating genomic and Sanger-sequencing data has great potential to capitalize on existing datasets with broad taxon sampling and to increase branch support, but also poses challenges^34^. For instance, many phylogenomic studies are restricted to small numbers of taxa^31^,^33^,^35^, and integration with broad, but shallow datasets will then introduce extensive levels of missing data^34^. Integration of genomic and Sanger-sequencing data matrices in phylogenetic reconstruction has rarely been explored^34^.

Transcriptomes have the potential to provide hundreds to thousands of protein-coding markers for phylogenetic analyses. They have been widely applied to and proven valuable in resolving ancient divergences in various insect groups with focus on interordinal and order-level relationships^30^,^36^,^37^,^38^. Here, we utilize transcriptomic data (370 loci) to determine relationships across the backbone of Reduvioidea, employ a “supermatrix” method to combine transcriptomic and ribosomal DNA data, and demonstrate the feasibility of integrating genomic and Sanger-sequencing data to produce a robust phylogenetic framework for a superfamily-level clade while exploring the evolution of raptorial legs within assassin bugs.

## Materials and Methods

### Taxon sampling

In total, 52 terminals are included, 48 of which are Reduvioidea (47 Reduviidae; 1 Pachynomidae) and 4 outgroups (see Table S1). Among them, transcriptomic data were obtained from 4 outgroups and 19 reduvioids sampled across major lineages of Reduvioidea. Taxon sampling covers all major raptorial leg types within the Reduvioidea, allowing for examination of the evolution of raptorial legs.

### Transcriptomic data

RNA extraction and purification of specimens were conducted using RNAlater preserved or liquid nitrogen frozen specimens (head and thorax for large, whole body for small specimens) and standard kits (e.g., RNeasy Fibrous Tissue, MasterPure^TM^ RNA Purification) in the Weirauch, Hwang/Meier, and Johnson labs. Quality and quantity of purified RNA were checked using an Agilent 2100 Bioanalyzer. cDNA library preparation and sequencing were completed at the W.M. Keck Center (University of Illinois) and AITBiotech PTE LTD (Singapore) using an Illumina HiSeq platform and paired-end 100-bp chemistry. Eight samples were multiplexed in one flow cell lane producing ~50,000,000 paired-end 100-bp raw reads (Table S2). The Illumina data were trimmed and then assembled in CLC Genomics Workbench 7.5.1 (http://www.clcbio.com) using a *de novo* assembly method. CLC Genomics Workbench stands out in sequence assemblers by its speed, accuracy, and user-friendly interface^39^. For quality trimming, the quality score limit was set to 0.001, a maximum of two ambiguities per read were allowed, and reads with length less than 30 bp were discarded. The *de novo* assembly was carried out using the automatic word and bubble size and limiting the minimum contig length to 300 bp.

Ortholog prediction analysis was carried out using HaMStR v13.2.2^40^ and a pre-compiled Insecta core-ortholog set with 1902 genes found in eight taxa (*Drosophila melanogaster* [Diptera], *Aedes aegypti* [Diptera], *Anopheles gambiae* [Diptera], *Tribolium castaneum* [Coleoptera], *Apis mellifera* [Hymenoptera], *Nasonia vitripennis* [Hymenoptera], *Acyrthosiphon pisum* [Hemiptera], *Daphnia pulex* [Crustacea]). The species most closely related to Reduviidae, the aphid *Acyrthosiphon pisum*, was assigned as the reference species for blast searches. The e-value cut-off for both HMM and blast searches was set to 1e^−20^, and the “representative” option was used to keep only one sequence showing the highest similarity to the core ortholog sequence in the reference species. To minimize the effect of missing data of the transcriptomic dataset on phylogenetic reconstruction, we only retained the 734 genes that cover all 23 taxa for subsequent phylogenomic analyses.

Alignments of the amino acid (AA) sequences of the orthologous genes were conducted using MAFFT v7.205^41^ with the G-INS-i algorithm. Alignment masking was performed on each orthologous gene using ALISCORE v2.0^42^ and ALICUT v2.3^43^ with default settings. The alignments of orthologous genes were manually checked and the genes with apparent paralogs were removed from subsequent analyses. After alignment masking, the orthologous genes with <100 AA or with at least one taxon containing only missing data were also removed from subsequent analyses. The remaining 466 orthologous genes were concatenated with FASconCAT v1.0^44^. The phylogenetic informative subset was then filtered using MARE v0.1.2-rc^45^ with d = 1 (other settings as default), which retained 370 orthologous genes for phylogenetic reconstruction. The partition scheme and model for each partition were optimized using PartitionFinder v1.1.1^46^ for the 370-gene AA matrix. The relaxed clustering algorithm was applied and each orthologous gene was treated as one subset in the initial data blocks.

### Ribosomal DNA data

Partial 16S, 18S, and 28S ribosomal DNA sequences for the 23 taxa with transcriptomic data were retrieved from their transcriptomic assemblies using BLAST + v2.2.29^47^ and the corresponding sequences of *Arilus cristatus* (Linnaeus) as queries. The 16S, 18S and 28S rDNA sequences of *Arilus cristatus* used for retrieving the rDNA sequences from the transcriptomic data were downloaded from GenBank (16S: FJ230402; 18S: FJ230477; 28S: FJ230558 + FJ230636 + FJ230715). The top-hit contig was selected and then aligned with the corresponding sequence of *Arilus cristatus* to trim off the unaligned regions from both ends. Ribosomal DNA sequences for other taxa were obtained from GenBank (see Table S1). Each ribosomal gene was then aligned using MAFFT with the G-INS-i algorithm.

### Phylogenetic tree inference

Phylogenetic reconstruction analyses were conducted on various matrices using the maximum likelihood (ML) criterion: the rDNA matrix with 52 taxa (R), the 370-gene AA matrix with 23 taxa (T), and the combined rDNA and 370-gene AA matrix with 52 taxa (T + R). Matrix completeness was calculated as the proportion of non-gap sites in the matrix. The ML analyses were performed in RAxML v.8.2.4^48^,^49^ via the IIGB Biocluster at the University of California, Riverside. The R dataset was divided into three partitions (16S, 18S and 28S) with a GTR + GAMMA model for each partition. The T dataset was divided into 288 partitions (each with the optimized AA substitution matrix by PartitionFinder and a GAMMA model of rate heterogeneity). The T + R dataset was divided into 291 partitions (288 partitions for the AA data plus 3 partitions for the rDNA data) with each partition having a GAMMA model of rate heterogeneity. The ML tree searches for each dataset were conducted using 50 distinct random trees. Non-parametric bootstrap analyses were completed to assess node support (1,000 replicates each for the R and T datasets; 500 replicates for the T + R dataset). Bootstrap convergence tests were conducted in RAxML (using the “–I autoMRE” option) on the bootstrap results from the R, T and T + R datasets to check if replicates were sufficient to obtain stable support values. The R dataset converged at 300 replicates, T at 50 replicates, and T + R at 300 replicates.

In addition, the 370-gene AA dataset was analyzed under a coalescent model to accommodate potential gene tree heterogeneity and discordance while also testing the robustness of our dataset under different analytical methods. The best-fitting model of protein evolution for each of the 370 AA genes was optimized using ProtTest v3.2^50^. ML analyses were performed on each AA gene in RAxML using the AA substitution matrix selected by ProtTest and a GAMMA model of rate heterogeneity. A hundred bootstrap replicates were completed for each of the 370 genes, which were imported into STAR^51^ to estimate the species tree.

### Evolution of raptorial legs

Six characters that represent different types of raptorial legs were scored based on observations of museum specimens or published data^20^,^22^: fossula spongiosa on tibia (ch. 1), tibia and femur modified into chela or subchela (ch. 2), armature (e.g., processes, setose tubercles, etc.) on trochanter (ch. 3), femur (ch. 4) and/or tibia (ch. 5), and sticky substances on forelegs (ch. 6). These features were scored as distinct characters with states defined as absent (0) and present (1). For the sticky substance character, the states were defined as absent (0), exogenous (1) and endogenous (2) to further distinguish the origins of the sticky substances on the legs^22^. This character was previously coded as “ambiguous” for *Pyrrhosphodrus amazonus* (Champion)^22^, but is here treated as “exogenous” to capture the exogenous sticky trap documented for closely related species, which were not included in this study. To test the correlation between the presence of a fossula spongiosa and any other modification on the forelegs, we also included another character (ch. 7) summarizing all modifications on the forelegs other than the fossula spongiosa that may assist in gripping prey: absent (0); present (1, at least one of the above modifications other than fossula spongiosa exists).

The evolution of each raptorial leg feature was reconstructed in Mesquite v2.75^52^ using ML (Mk1 model) and parsimony methods. Correlations between fossula spongiosa and each of the modifications (ch. 2–6), as well as any other modification (ch. 7), were tested using ML and Bayesian methods in BayesTraits v2^53^. In the correlation analyses, ch. 6 was transformed into a binary character with both exogenous and endogenous sticky substance categorized as present (1). The ML tree from the T + R dataset was used for all analyses. For the Bayesian method, the analyses were run for 11 million iterations, sampled every 1,000 iterations with the first 1 million iterations discarded as burn-in. The hyper prior approach was used to set up the prior distributions of the parameters of the evolutionary model (“HyperPriorAll exp 0 10”). Independent (4 parameters) and dependent (8 parameters) models were compared using likelihood ratio tests for ML results and Bayes factors for Bayesian results. For likelihood ratio tests, when the likelihood ratio (LR), computed as 2 × (log[likelihood (dependent model)] − log[likelihood (independent model)]), is greater than 9.488 (P < 0.05, df = 4), we concluded that the two characters, were coevolved^54^. For Bayes factors (BF), computed as 2 × (log[harmonic mean (dependent model)] − log[harmonic mean(independent model)]), values ≥6 were taken as strong evidence for correlated evolution (see BayesTrait Manual).

## Results and Discussion

Our phylogenomic analysis based on transcriptomic data of Reduvioidea aims at resolving the backbone phylogeny of assassin bugs. Our results prove the efficacy of this approach to resolve divergences within a superfamily and family that are more recent than those that have typically been the focus of previous studies (i.e., between and within orders). To minimize the systematic error that grows with dataset size and potential effects of missing data^55^, transcriptomic data were trimmed and filtered to remove misaligned and phylogenetically uninformative regions, and only genes present in all taxa were retained for analyses. The transcriptomic dataset (23 taxa; 11 subfamilies) contains 370 genes (~180,000 AA) with an overall matrix completeness of 90.95%. The inferred maximum likelihood (ML) tree (Fig. S1A) is well-resolved and well-supported, and the topology is largely congruent with the species tree derived from our STAR analysis (Fig. S1B; but see differing placement of *Rhiginia ruficoria* Maldonado and *Acanthaspis quadriannulata* Stål), supporting the notion that this backbone phylogeny is robust under drastically different analytical methods. The Peiratinae, known for their painful bites, are supported as the sister clade to all other Higher Reduviidae, while the cryptic, subcorticulous Reduviinae and Physoderinae are close relatives of the often aposematic, plant-dwelling Harpactorinae.

**Figure 2:**
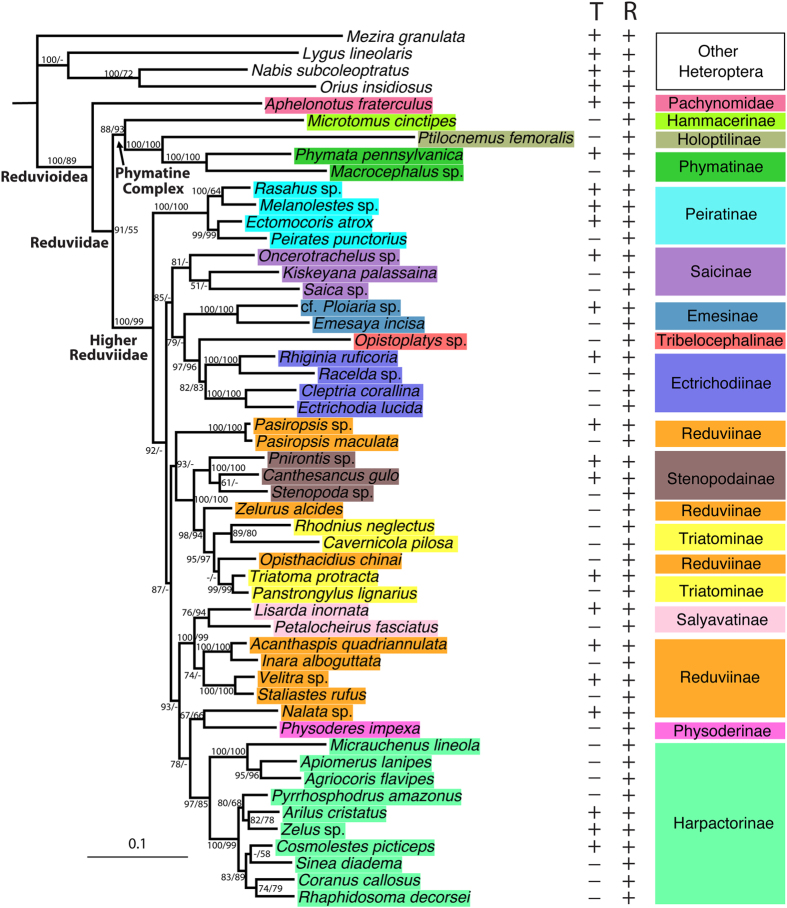
The best tree from the ML analysis (lnL = −1875326.55) of the combined matrix of transcriptomic (T; 23 transcriptomes; 370 genes; 179, 367 amino acids; + present,-absent) and rDNA (R; 16S, 18S, 28S; 3,101 nucleotides) datasets. Numbers on clades indicate support from RAxML bootstrap analysis with the first referring to the T + R dataset and the second to the R dataset; “−” denotes bootstrap support <50%.

To allow for tests of the raptorial leg evolution based on a phylogeny that includes representatives of all raptorial leg types, we assembled a supermatrix (52 taxa) by concatenating the transcriptomic dataset (T) with a ribosomal DNA dataset (R; 16S, 18S and 28S; ~3,000 bp), resulting in a matrix (T + R) with 40.92% completeness. Despite a high proportion of missing data, the ML tree inferred from the T + R matrix (Fig. 2) is highly resolved with well-supported deep and shallow nodes. The backbone relationships of Reduvioidea in the T + R topology (Fig. 2) are congruent with those in the T topology (RAxML best tree, Fig. S1A), and the well-supported branches among the T + R, T and R (Fig. S2) phylogenies are largely compatible. This indicates that the well-resolved and well-supported phylogeny from the T + R dataset is unlikely due to missing data artifacts. The monophyly of Reduvioidea, which was proposed based on morphological characters^56^,^57^, is corroborated for the first time based on molecular data (ML bootstrap support [bs] = 100% [T + R]; 100% [T]; 89% [R]). The Phymatine Complex of reduviid subfamilies that comprises, amongst others, the ant-luring feather-legged bugs (Holoptilinae) and cryptic ambush bugs (Phymatinae) is supported (bs = 88% [T + R]; 93% [R]), as are the Higher Reduviidae that contain all remaining assassin bugs (bs = 100% [T + R]; 100% [T]; 99% [R]). Most of the subfamilies or groups of subfamilies within the Higher Reduviidae that were recovered in prior phylogenies are supported in our analyses, e.g., Peiratinae (bs = 100% [T + R]; 100% [T]; 100% [R]), and a clade containing Stenopodainae, *Zelurus*, *Opisthacidius* and the blood-feeding Triatominae (bs = 100% [T + R]; 100% [R]). The four included species of kissing bugs (Triatominae), representing 3 of the 5 tribes, are rendered paraphyletic by *Opisthacidius* (Fig. 2). This grouping has low support (bs <50% in both T + R and R), thus precluding new insights into the debated relationships and evolution of Chagas disease vectors^2^,^27^,^29^,^58^. Reduviinae are corroborated to be polyphyletic with members spread among at least four different clades.

However, most of the dramatic improvements of the T + R topology over prior phylogenies lie in the deeper nodes within the Higher Reduviidae (Fig. 2). The phylogenetic placement of Peiratinae, previously proposed as sister to the remaining Higher Reduviidae^2^ with bs <50%, is now strongly supported (bs for remaining Higher Reduviidae = 92% [T + R]). A clade that comprises the millipede assassin bugs (Ectrichodiinae) and the spider-web inhabiting thread-legged bugs (Emesinae), amongst other subfamilies (Saicinae, Tribelocephalinae), was moderately supported (bs = 85% [T + R]), corroborating a hypothesis based on weak morphological support^27^. The remaining Higher Reduviidae fall into two clades. One comprises the kissing bugs and related groups (bs = 93% [T + R]: Triatominae, Stenopodainae, the *Zelurus* and *Pasiropsis* clades of Reduviinae). The other comprises the termite assassins (Salyavatinae), Harpactorinae and other related groups (bs = 93% [T + R]: *Acanthaspis*, *Nalata* and *Velitra* clades of Reduviinae; Physoderinae). These results for the first time provide structure to the backbone of Higher Reduviidae.

**Figure 3:**
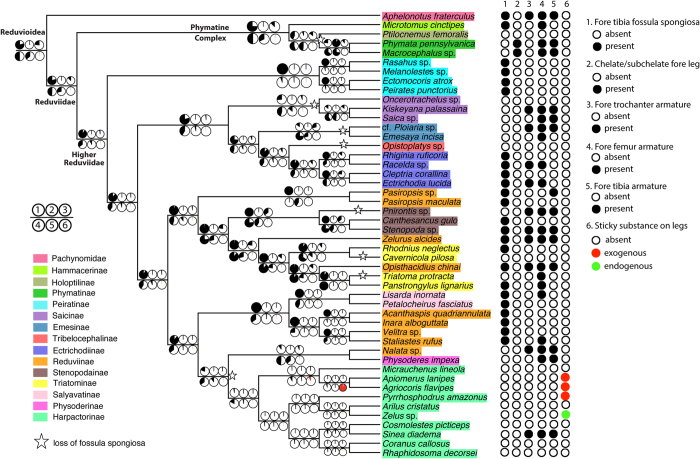
Summary of the maximum likelihood ancestral state reconstructions for raptorial leg features. The tree shown is the ML best tree from the T + R dataset with non-reduvioid taxa trimmed off. The pie charts along the branches illustrate the likelihood for the ancestral states reconstructed in Mesquite 2.75. Scoring of each leg feature for taxa is indicated in the circles after the taxon names.

When evaluating the evolution of raptorial legs across Reduvioidea based on the T + R phylogeny, we find that the fossula spongiosa, which has long been implicated to be associated with raptorial behaviors in assassin bugs^16^,^59^,^60^, was indeed present in the last common ancestors of Reduvioidea and Reduviidae. This feature was then lost multiple times within Reduviidae (Fig. 3). Reconstructions of the forefemoral armature at the base of Reduvioidea and Reduviidae are ambiguous. It remains unclear if strong spines and processes, as seen in many Reduvioidea, are plesiomorphic for the clade and were subsequently lost, or if they evolved independently in various reduviid lineages. In contrast, other raptorial leg modifications likely evolved within assassin bugs. These modifications include the unique chelate and subchelate legs that evolved within ambush bugs, but also modifications that evolved multiple times independently in different lineages, such as the armature on the trochanters and tibiae or the use of sticky substances to enhance prey capture. To test if the evolution of alternative raptorial legs in Reduvioidea is correlated with the loss of the fossula spongiosa, we conducted correlation tests using BayesTraits. The dependent model for each character pair was found to be not significantly better, if not worse, than the independent model (both likelihood ratio and Bayes Factor <4; Table S3). We therefore conclude that these alternative raptorial leg traits likely evolved independently from the loss of the fossula spongiosa, contradicting previous hypotheses that suggested correlated evolution^18^,^22^.

Other morphological and behavioral factors have almost certainly influenced the evolution of raptorial legs, ranging from prey specializations to the toxicity of the paralytic saliva. Certain assassin bugs lack raptorial legs altogether. For example, some Harpactorinae, Holoptilinae, and Tribelocephalinae possess slender legs without armature, fossula spongiosa, or sticky substances. However, some of these taxa show behaviors relaxing the constraint of tightly holding prey before the saliva immobilizes the prey item. The most prominent example of this behavior is seen in certain Holoptilinae, which have been reported to sedate ants with fluids released from an abdominal trichome^10^,^15^. Forefemoral armature observed in some vertebrate-blood feeding Triatominae (Fig. 3) may be retained plesiomorphic features reflecting the evolution of these bugs from typical predators. Interestingly, numerous species of Triatominae still possess a fossula spongiosa (Fig. 3). Lent and Wygodzinsky^3^ reported that the fossula is sexually dimorphic in Triatominae (i.e., larger in males than females), indicating that the function of this structure may have shifted from a role in prey capture to one associated with mating.

Salivary toxicity may be another factor influencing the evolution of raptorial leg features. Saliva with higher toxicity may paralyze and kill prey faster and could remove constraints on raptorial legs. Studies have shown that assassin bugs without fossula spongiosa immobilize their prey faster than those with fossula spongiosa^12^, supporting this hypothesis. We conclude that understanding morphological adaptations to prey capture in assassin bugs will benefit from additional research. Studies should focus on carefully teasing apart the complex relationships between prey specialization, predatory behavior, and function of salivary compounds.

## Additional Information

**Accession numbers**: rDNA sequences from the transcriptomic data have been deposited in GenBank with the accession numbers KT231814-KT231896. The matrices, corresponding partition files and gene trees are available from the Dryad Digital Repository: http://dx.doi.org/10.5061/dryad.1n6s9.

**How to cite this article**: Zhang, J. *et al.* Evolution of the assassin's arms: insights from a phylogeny of combined transcriptomic and ribosomal DNA data (Heteroptera: Reduvioidea). *Sci. Rep.* **6**, 22177; doi: 10.1038/srep22177 (2016).

## Supplementary Material

Supplementary Information

## Figures and Tables

**Figure 1:**
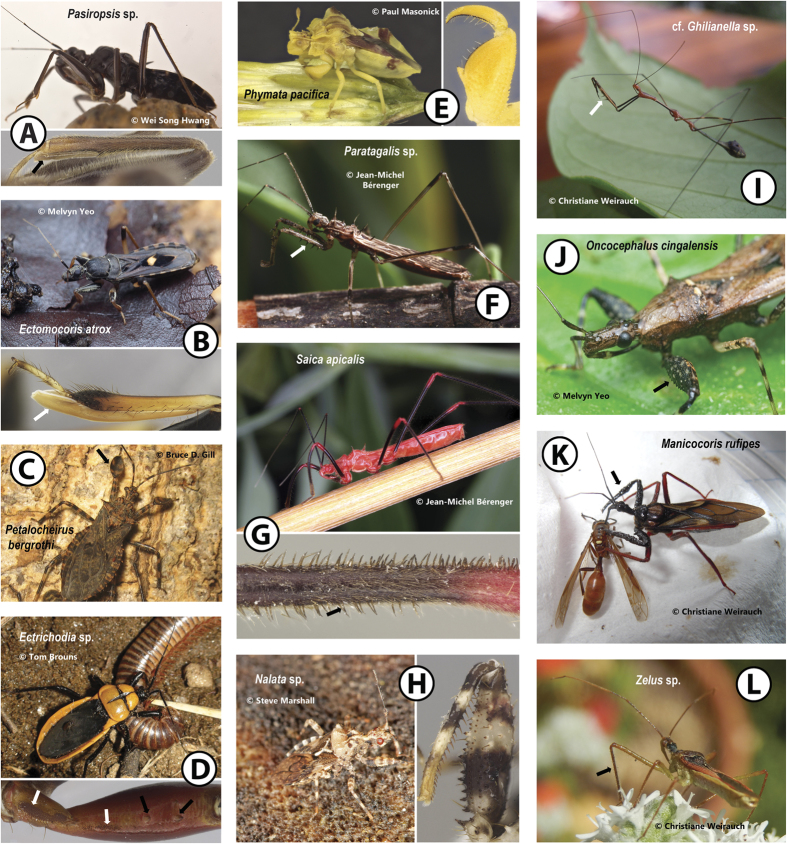
Modifications on the forelegs in Reduviidae. (**A**) *Pasiropsis* sp. with black arrow showing the moderately-sized fossula spongiosa on the foretibia; (**B**) *Ectomocoris atrox* with white arrow showing elongate fossula spongiosa on foretibia; (**C**) *Petalocheirus bergrothi* with black arrow showing enlarged foretibia; (**D**) *Ectrichodia* sp. with white arrows showing small papillae on foretrochanter and -femur, and black arrows showing small processes on forefemur; (**E**) *Phymata pacifica* with foreleg modified into a subchela; (**F**) *Paratagalis* sp. with white arrow showing pronounced spines on foreleg; (**G**) *Saica apicalis* with black arrow showing modified setae on foreleg; (**H**) *Nalata* sp. with pronounced armature (spines and processes) on foreleg; (**I**) cf. *Ghilianella* sp. with white arrow showing pronounced spines on foreleg; (**J**) *Oncocephalus cingalensis* with black arrow showing small processes on forefemur; (**K**) *Manicocoris rufipes* with black arrow showing the foreleg coated by sticky substance from plant resin; (**L**) *Zelus* sp. with black arrow showing the foreleg coated with sticky substance produced by sticky glands. Living assassin bug photos of (**B**) and (**J**) taken by Melvyn Yeo, (**C**) by Bruce D. Gill, (**D**) by Tom Brouns, (**E**) by Paul Masonick, (**F**) and (**G**) by Jean-Michel Bérenger, (**H**) by Steve Marshall.
