# Supplementary material for: Evolution of the assassin’s arms: insights from a phylogeny of combined transcriptomic and ribosomal DNA data (Heteroptera: Reduvioidea)
Source: Sci Rep. 2016 Feb 26;6:22177. doi: 10.1038/srep22177 (PMC4768186; doi:10.1038/srep22177)
Supplement: Supplementary Information [file srep22177-s1.pdf]

Supplementary information

Evolution of the assassin's arms: insights from a phylogeny of combined transcriptomic and ribosomal DNA data (Heteroptera: Reduvioidea)

Junxia Zhang[1*], Eric R.L. Gordon[1], Michael Forthman[1], Wei Song Hwang[2], Kim Walden[3], Daniel R. Swanson[3], Kevin P. Johnson[3], Rudolf Meier[2], Christiane Weirauch[1]

1. Department of Entomology, University of California, Riverside, Riverside, CA 92521, USA

2. Lee Kong Chian Natural History Museum, Department of Biological Sciences, National University of Singapore, 117377, Singapore

3. Illinois Natural History Survey, Prairie Research Institute, University of Illinois, Champaign, IL 61820, USA

* Corresponding author. E-mail: jxzhang1976@gmail.com

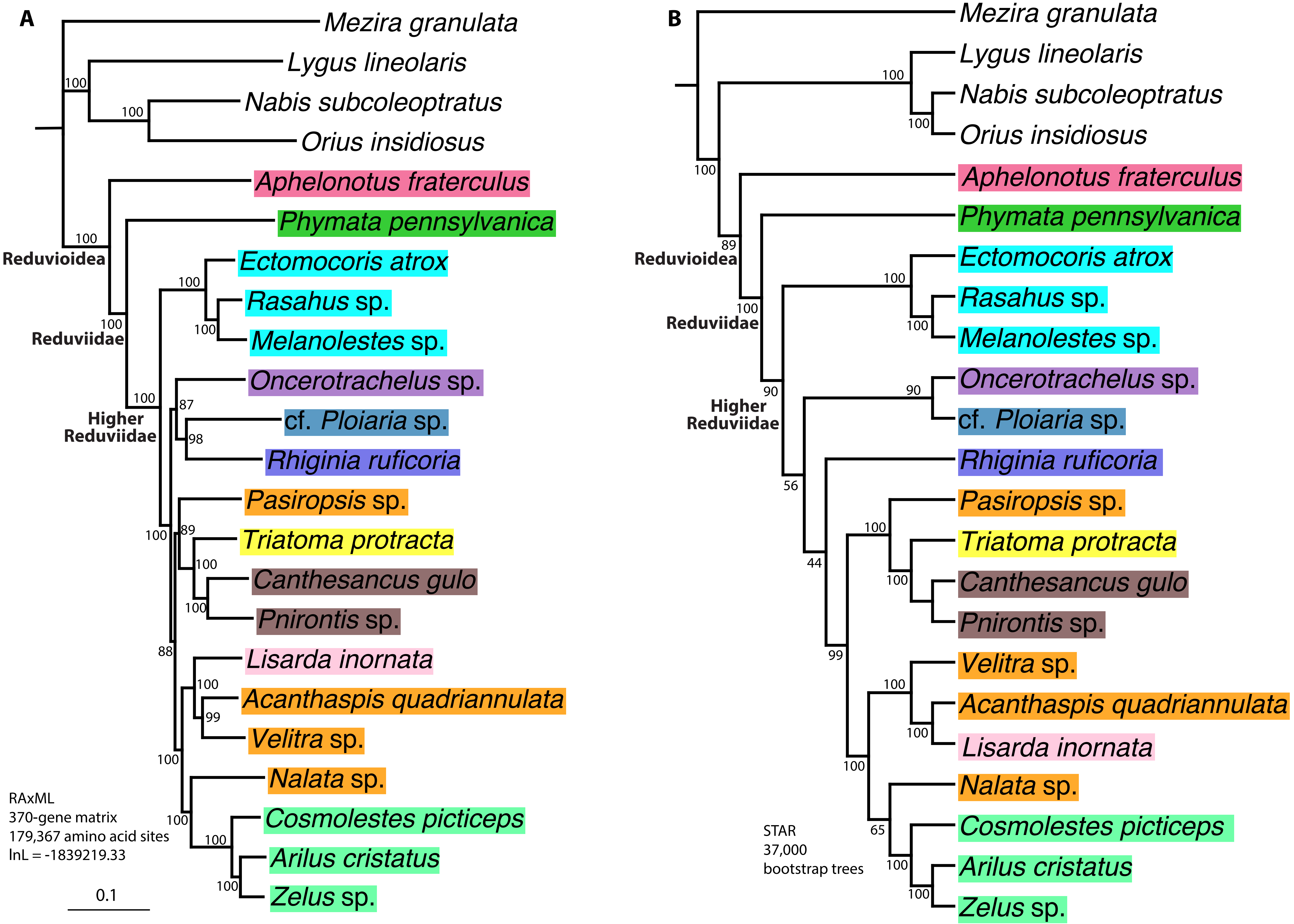

Figure S1. Phylogenies inferred from RAxML (1A) and STAR (1B) based on the transcriptomic dataset (23 transcriptomes; 370 genes).

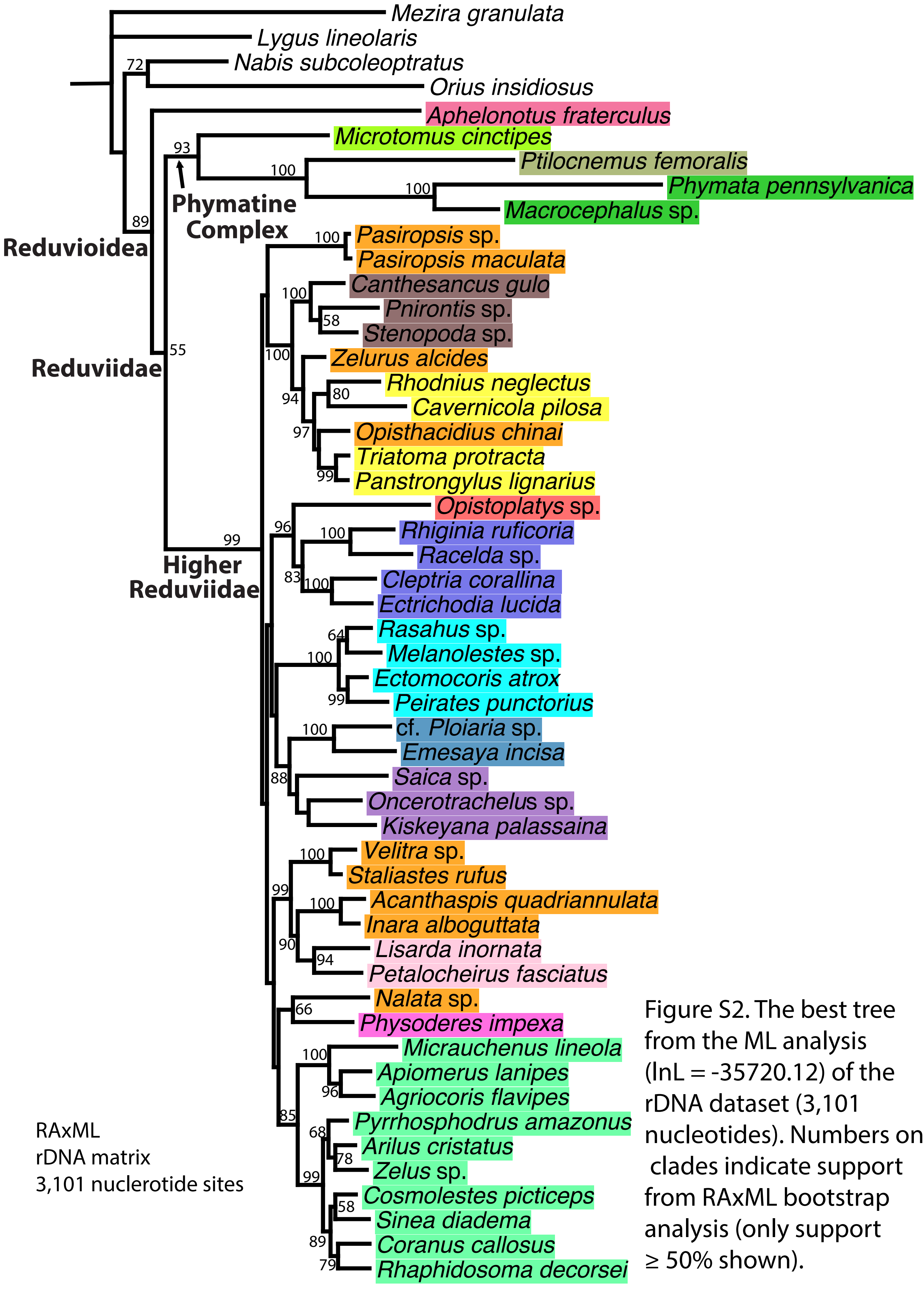

Figure S2. The best tree from the ML analysis (lnL = -35720.12) of the rDNA dataset (3,101 nucleotides). Numbers on clades indicate support from RAxML bootstrap analysis (only support ≥ 50% shown).

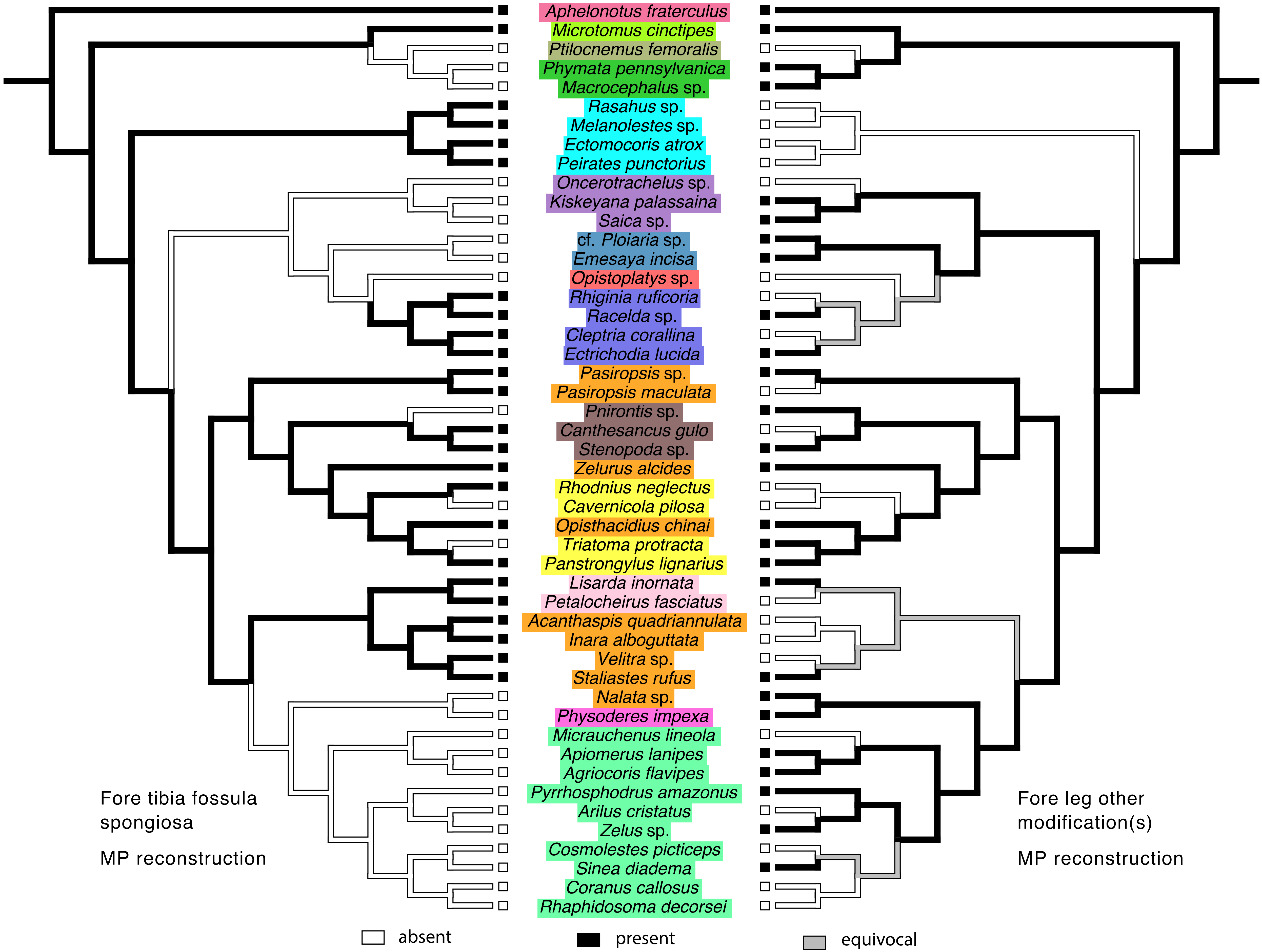

Figure S3. The evolution of the fossula spongiosa (left) and any other leg modification (right) in Reduvioidea. The tree shown is the ML best tree from the T+R dataset with non-reduvioid taxa trimmed off. Ancestral states reconstructed using parsimony in Mesquite 2.75.

Table S1. List of species sampled in this study. Species names, specimen code, collection localities, transcriptome and rDNA gene information (GenBank accession number). (*) denotes previously published sequences.

Infraorder	Family	Subfamily	Taxon	Sample Code	Locality	Transcriptome	16S	18S	28SD2	28SD3-5
Cimicomorpha	Reduviidae	Ectrichodiinae	*Cleptria corallina* Villiers	RCW014	Guinea-Bissau	no	*FJ230388	*FJ230462	*FJ230543	*FJ230621; *FJ230700
Cimicomorpha	Reduviidae	Ectrichodiinae	*Ectrichodia lucida* Lepelletier & Serville	RCW013	Guinea-Bissau	no	*FJ230387	*FJ230461	*FJ230542	*FJ230620; *FJ230699
Cimicomorpha	Reduviidae	Ectrichodiinae	*Racelda* sp.	RCW041	French Guiana: Approuague-Kaw	no	*FJ230398	*FJ230472	*FJ230553	*FJ230631; *FJ230710
Cimicomorpha	Reduviidae	Ectrichodiinae	*Rhiginia ruficoria* Maldonado	RCW3947	Argentina: Santiago del Estero	yes	**KT231822**	**KT231845**	**KT231864**	**KT231885**
Cimicomorpha	Reduviidae	Emesinae	*Emesaya incisa* (McAtee & Malloc)	RCW282	USA: California	no	*FJ230436	*FJ230515	*FJ230598	*FJ230672; *FJ230751
Cimicomorpha	Reduviidae	Emesinae	cf. *Ploiaria* sp.	RCW3916	Dominican Republic: La Altagracia	yes	**KT231821**	**KT231844**	**KT231863**	**KT231884**
Cimicomorpha	Reduviidae	Hammacerinae	*Microtomus cinctipes* (Stål)	RCW141	Nicaragua	no	*FJ230411	*FJ230491	no	*FJ230649; *FJ230728
Cimicomorpha	Reduviidae	Harpactorinae	*Agriocoris flavipes* (Fabricius)	RCW132	French Guiana: Cayenne	no	*JQ942236	*FJ230488	*FJ230569	*FJ230646; *FJ230725
Cimicomorpha	Reduviidae	Harpactorinae	*Apiomerus lanipes* (Fabricius)	RCW281	Argentina: Santiago del Estero	no	*FJ230435	*FJ230514	*FJ230597	*FJ230671; *FJ230750
Cimicomorpha	Reduviidae	Harpactorinae	*Arilus cristatus* (Linnaeus)	P025	USA: Illinois	yes	**KT231832**	**KT231855**	**KT231873**	**KT231895**
Cimicomorpha	Reduviidae	Harpactorinae	*Coranus callosus* Stål	RCW244	Australia: Western Australia	no	*FJ230433	*FJ230511	*FJ230594	*FJ230669; *FJ230748

Cimicomorpha	Reduviidae	Harpactorinae	*Cosmolestes picticeps* (Stål)	ROU010	Singapore: Mandai	yes	**KT231831**	**KT231854**	**KT231872**	**KT231894**
Cimicomorpha	Reduviidae	Harpactorinae	*Micrauchenus lineola* (Fabricius)	RCW035	French Guiana	no	*FJ230397	*FJ230471	*FJ230552	*FJ230630; *FJ230709
Cimicomorpha	Reduviidae	Harpactorinae	*Pyrrhosphodrus amazonus* (Champion)	RCW031	French Guiana: Montsinery	no	*FJ230396	*FJ230470	*FJ230551	*FJ230629; *FJ230708
Cimicomorpha	Reduviidae	Harpactorinae	*Rhaphidosoma decorsei* Jeannel	RCW017	Senegal: Thies	no	*FJ230390	*FJ230464	*FJ230545	*FJ230622; *FJ230701
Cimicomorpha	Reduviidae	Harpactorinae	*Sinea diadema* Caudell	RCW108	Mexico: Chihuahua	no	*FJ230408	*FJ230485	*FJ230566	*FJ230644; *FJ230723
Cimicomorpha	Reduviidae	Harpactorinae	*Zelus* sp.	RCW3869	USA: California	yes	**KT231833**	**KT231856**	**KT231874**	**KT231896**
Cimicomorpha	Reduviidae	Holoptilinae	*Ptilocnemus femoralis* Horvath	RCW220	Australia: South Australia	no	*FJ230431	*FJ230509	*FJ230591	*FJ230667; *FJ230746
Cimicomorpha	Reduviidae	Peiratinae	*Ectomocoris atrox* (Stål)	ROU006	Singapore: Seletar	yes	**KT231819**	**KT231842**	**KT231861**	**KT231882**
Cimicomorpha	Reduviidae	Peiratinae	*Melanolestes* sp.	RCW3946	Argentina: Misiones	yes	**KT231818**	**KT231841**	**KT231860**	**KT231881**
Cimicomorpha	Reduviidae	Peiratinae	*Peirates punctorius* (Stål)	RCW216	Australia: New South Wales	no	*FJ230430	*FJ230508	*FJ230590	*FJ230666; *FJ230745
Cimicomorpha	Reduviidae	Peiratinae	*Rasahus* sp.	RCW3821	Costa Rica	yes	**KT231817**	**KT231840**	**KT231859**	**KT231880**
Cimicomorpha	Reduviidae	Phymatinae	*Macrocephalus* sp.	RCW128	Mexico: Chihuahua	no	*FJ230409	*FJ230487	*FJ230568	*FJ230645; *FJ230724
Cimicomorpha	Reduviidae	Phymatinae	*Phymata pennsylvanica* Handlirsch	INHS-780,184	USA: Illinois	yes	**KT231816**	**KT231839**	**KT231858**	**KT231879**
Cimicomorpha	Reduviidae	Physoderinae	*Physoderes impexa* (Distant)	RCW1572	Vietnam: Vinh Phuc	no	*JQ897830	*JQ897591	*JQ897662	*JQ897748
Cimicomorpha	Reduviidae	Reduviinae	*Acanthaspis quadriannulata* Stål	ROU007	Singapore: Nee Soon	yes	**KT231823**	**KT231846**	**KT231865**	**KT231886**
Cimicomorpha	Reduviidae	Reduviinae	*Inara alboguttata* Stål	RCW1164	Thailand; Nakhon Nayok	no	*JQ897801	*JQ897561	*JQ897640	*JQ897718

Cimicomorpha	Reduviidae	Reduviinae	*Nalata* sp.	RCW3822	Costa Rica	yes	**KT231826**	**KT231849**	**KT231868**	**KT231889**
Cimicomorpha	Reduviidae	Reduviinae	*Opisthacidius chinai* Lent & Wygodzinsky	RCW1285	Peru	no	*JQ897819	*JQ897580	*JQ897652	*JQ897737
Cimicomorpha	Reduviidae	Reduviinae	*Pasiropsis maculata* Distant	RCW810	Thailand	no	*JQ897825	*JQ897586	*JQ897658	*JQ897743
Cimicomorpha	Reduviidae	Reduviinae	*Pasiropsis* sp.	ROU009	Singapore: Nee Soon	yes	**KT231824**	**KT231847**	**KT231866**	**KT231887**
Cimicomorpha	Reduviidae	Reduviinae	*Staliastes rufus* (Laporte)	RCW690	Laos	no	*JQ897842	*JQ897604	*JQ897673	*JQ897758
Cimicomorpha	Reduviidae	Reduviinae	*Velitra* sp.	ROU012	Singapore: Rifle Range Road	yes	**KT231825**	**KT231848**	**KT231867**	**KT231888**
Cimicomorpha	Reduviidae	Reduviinae	*Zelurus alcides* (Stål)	RCW1571	Costa Rica: Heredia	no	*JQ897855	*JQ897615	*JQ897686	*JQ897771
Cimicomorpha	Reduviidae	Saicinae	*Kiskeyana palassaina* Weirauch & Forero	RCW010	Dominican Republic	no	no	*FJ230460	*FJ230541	*FJ230619; *FJ230698
Cimicomorpha	Reduviidae	Saicinae	*Oncerotrachelus* sp.	RCW3825	Costa Rica	yes	**KT231820**	**KT231843**	**KT231862**	**KT231883**
Cimicomorpha	Reduviidae	Saicinae	*Saica* sp.	RCW042	French Guiana: Approuague-Kaw	no	*FJ230399	*FJ230473	*FJ230554	*FJ230632; *FJ230711
Cimicomorpha	Reduviidae	Salyavatinae	*Lisarda inornata* (Walker)	ROU008	Singapore: Sime track	yes	**KT231830**	**KT231853**	no	**KT231893**
Cimicomorpha	Reduviidae	Salyavatinae	*Petalocheirus fasciatus* Distant	RCW812	Thailand; Nakhon Si Thammarat	no	*KP236992	*KP236966	*KP236940	*KP237023
Cimicomorpha	Reduviidae	Stenopodainae	*Canthesancus gulo* Stål	ROU005	Singapore: Seletar	yes	**KT231828**	**KT231851**	**KT231870**	**KT231891**
Cimicomorpha	Reduviidae	Stenopodainae	*Pnirontis* sp.	RCW3945	Argentina: Misiones	yes	**KT231829**	**KT231852**	**KT231871**	**KT231892**
Cimicomorpha	Reduviidae	Stenopodainae	*Stenopoda* sp.	RCW154	Nicaragua: Granada	no	*FJ230414	*FJ230493	*FJ230574	*FJ230651; *FJ230730

Cimicomorpha	Reduviidae	Triatominae	*Cavernicola pilosa* Barber	RCW2847	Nicaragua: Rio San Juan	no	*JQ897785	*JQ897550	*JQ897627	*JQ897704
Cimicomorpha	Reduviidae	Triatominae	*Panstrongylus lignarius* (Walker)	RCW1813	French Guiana: Roura	no	*JQ897823	*JQ897584	*JQ897656	*JQ897741
Cimicomorpha	Reduviidae	Triatominae	*Rhodnius neglectus* Lent	RCW1573	Ecuador: Orellana	no	*JQ897839	*JQ897601	*JQ897670	*JQ897755
Cimicomorpha	Reduviidae	Triatominae	*Triatoma protracta* Uhler	ROU011	USA: California	yes	**KT231827**	**KT231850**	**KT231869**	**KT231890**
Cimicomorpha	Reduviidae	Tribelocephalinae	*Opistoplatys* sp.	RCW1592	Brunei: Belait	no	*JQ897851	*JQ897612	*JQ897682	*JQ897767
Cimicomorpha	Pachynomidae	Aphelonotinae	*Aphelonotus fraterculus* Harris	RCW3830	Costa Rica: Guanacaste	yes	**KT231815**	**KT231838**	**KT231857**	**KT231878**
Cimicomorpha	Miridae	Mirinae	*Lygus lineolaris* (Palisot de Beauvois)	INHS-780,185	USA: Illinois	yes	*AY252649	**KT231835**	no	**KT231875**
Cimicomorpha	Nabidae	Nabinae	*Nabis subcoleoptratus* (Kirby)	INHS-780,193	USA: South Dakota	yes	no	**KT231836**	no	**KT231876**
Cimicomorpha	Anthocoridae	Oriini	*Orius insidiosus* (Say)	INHS-780,194	USA: Illinois	yes	**KT231814**	**KT231837**	no	**KT231877**
Pentatomomorpha	Aradiidae	Mezirinae	*Mezira granulata* (Say)	INHS-780,195	USA: Illinois	yes	*AY252694	**KT231834**	no	no

Table S2. Illumina RNA-seq, quality trimming, de novo assembly, and HaMStR ortholog prediction results, showing the number of paired-end raw reads generated for each library, the number of filtered raw reads after quality trimming for de novo assembly, the number of assembled contigs with their average length and N50, the number of ortholog genes (OGs) from HaMStR, and the completeness in the concatenated matrix (calculated as the proportion of non-gap sites in the matrix). 370 protein-coding genes that cover all 23 taxa are used for phylogenetic analyses.

Family	Subfamily	Taxon	# Raw Reads	# Filtered Reads	# Contigs	Averge Length of Contigs	N50 of Contigs	# OGs	Completeness (%)
Reduviidae	Phymatinae	*Phymata pennsylvanica* Handlirsch	59,164,110	55,152,329	28,686	929	1,302	1,617	90.2
Reduviidae	Peiratinae	*Ectomocoris atrox* (Stål)	60,708,978	57,693,491	23,489	1,134	1,708	1,671	96.4
Reduviidae	Peiratinae	*Melanolestes* sp.	45,434,656	43,563,840	21,523	978	1,369	1,687	94.8
Reduviidae	Peiratinae	*Rasahus* sp.	50,766,124	46,816,267	21,201	1,031	1,449	1,660	91.1
Reduviidae	Saicinae	*Oncerotrachelus* sp.	51,921,462	47,908,459	24,779	1,126	1,704	1,692	95.3
Reduviidae	Emesinae	cf. *Ploiaria* sp.	35,413,568	33,996,569	27,594	922	1,207	1,669	86.2
Reduviidae	Ectrichodiinae	*Rhiginia ruficoria* Maldonado	41,997,172	40,391,579	20,155	857	1,062	1,538	73.8
Reduviidae	Reduviinae	*Acanthaspis quadriannulata* Stål	36,274,778	34,535,692	21,613	1,062	1,511	1,582	91.4
Reduviidae	Reduviinae	*Pasiropsis* sp.	45,732,464	43,719,356	31,208	1,052	1,528	1,706	94.9
Reduviidae	Reduviinae	*Velitra* sp.	45,706,512	43,233,262	32,617	930	1,252	1,631	88.6
Reduviidae	Reduviinae	*Nalata* sp.	56,725,054	52,251,972	30,341	1,062	1,574	1,674	95.5
Reduviidae	Stenopodainae	*Canthesancus gulo* Stål	46,225,452	44,191,605	18,875	938	1,223	1,473	79.0
Reduviidae	Stenopodainae	*Pnirontis* sp.	46,197,746	44,427,838	26,371	923	1,237	1,671	88.8
Reduviidae	Salyavatinae	*Lisarda inornata* (Walker)	48,007,458	45,762,541	30,613	1,106	1,649	1,647	95.5
Reduviidae	Triatominae	*Triatoma protracta* Uhler	57,591,970	54,638,744	39,000	1,055	1,560	1,673	95.4
Reduviidae	Harpactorinae	*Arilus cristatus* (Linnaeus)	56,077,314	48,995,858	23,901	997	1,399	1,637	92.9
Reduviidae	Harpactorinae	*Zelus* sp.	55,174,874	51,198,146	27,330	1,080	1,588	1,683	96.3
Reduviidae	Harpactorinae	*Cosmolestes picticeps* (Stål)	42,559,608	40,613,067	24,166	1,109	1,590	1,655	95.7
Pachynomidae	Aphelonotinae	*Aphelonotus fraterculus* Harris	54,026,628	49,466,008	26,339	973	1,352	1,535	79.0
Miridae	Mirinae	*Lygus lineolaris* (Palisot de Beauvois)	49,691,658	45,520,824	29,487	1,155	1,787	1,692	95.6
Nabidae	Nabinae	*Nabis subcoleoptratus* (Kirby)	47,076,394	43,642,823	25,265	1,110	1,696	1,708	96.2
Anthocoridae	Oriini	*Orius insidiosus* (Say)	48,882,610	44,996,984	19,245	1,252	1,882	1,675	93.3
Aradiidae	Mezirinae	*Mezira granulata* (Say)	52,534,656	48,880,819	34,824	952	1,340	1,681	86.2

Table S3. Summary of correlation tests using Bayesian and maximum likelihood methods in BayesTraits, showing that the dependent model is not significantly better than the independent model for each character pair with BF<6 and LR<9.488 (p>0.2, d.f.=4).

Character Pair	MCMC			ML		
	log (harmonic mean)		Bayes Factor (BF)	log (likelihood)		Likelihood Ratio (LR)
	Dependent	Independent		Dependent	Independent	
1-2: Fossula Spongiosa-Chela/Subchela	-30.780	-30.119	-1.323	-24.370	-25.685	2.629
1-3: Fossula Spongiosa-Trochanter armature	-52.172	-48.497	-7.351	-45.779	-45.906	0.254
1-4: Fossula Spongiosa-Femur armature	-55.362	-55.644	0.565	-49.446	-51.161	3.430
1-5: Fossula Spongiosa-Tibia armature	-54.898	-54.143	-1.511	-48.294	-50.203	3.818
1-6: Fossula Spongiosa-Sticky trap	-37.449	-37.335	-0.228	-30.581	-32.359	3.555
1-7: Fossula Spongiosa-Any of other modification	-61.169	-59.890	-2.557	-53.281	-54.360	2.157
